# Efficacy and Safety of Duhuo Jisheng Decoction for Postmenopausal Osteoporosis: A Systematic Review and Meta-Analysis

**DOI:** 10.1155/2020/6957825

**Published:** 2020-09-15

**Authors:** Jinyu Li, Wei Wang, Guiyu Feng, Jian Du, Shengqian Kang, Zhe Li, Weifeng Zhu, Hongcai Shang

**Affiliations:** ^1^Key Laboratory of Chinese Internal Medicine of the Ministry of Education, Dongzhimen Hospital Affiliated to Beijing University of Chinese Medicine, Beijing 100700, China; ^2^Key Laboratory of Modern Chinese Medicine Preparation of Ministry of Education, Jiangxi University of Traditional Chinese Medicine, Nanchang, Jiangxi 330004, China; ^3^District 1 of Orthopedics, Dongzhimen Hospital, Beijing University of Chinese Medicine, Beijing, China; ^4^Beijing University of Chinese Medicine, Beijing, China

## Abstract

**Aim:**

To evaluate the effects and medication safety of Duhuo Jisheng Decoction (DHJSD) alone or as a combination therapy with other interventions on the related clinical index in postmenopausal osteoporosis condition.

**Methods:**

Search in CNKI, WanFang, CBM, VIP, PubMed, EMBASE, and Cochrane Library databases and randomized controlled trials where at least one group received any form of DHJSD for postmenopausal osteoporosis condition. Risk of bias was based on the Cochrane handbook, the quality of evidence was assessed by the GRADEpro online, and analyses were performed by RevMan 5.3 software.

**Results:**

Eight studies were enrolled with 650 participants. DHJSD alone or with other interventions had a significant effect on BMD of the lumbar spine (MD = 0.46, 95%CI (0.24, 0.68), *P* < 0.0001), E2 (SMD = 0.49, 95%CI (0.30, 0.68), *P* < 0.0001), and clinical effectiveness (OR = 5.07, 95%CI (3.07, 8.35), *P* < 0.0001). However, no effect at BGP (MD = −0.84, 95%CI (−1.69, 0.00), *P*=0.05) was seen.

**Conclusion:**

The pooled estimate suggested that DHJSD combined with conventional medical therapies has a certain clinical curative effect on postmenopausal osteoporosis. However, considering the unsatisfactory quality of included trials, more high-quality trials are needed to elucidate this issue.

## 1. Introduction

Postmenopausal osteoporosis (PMOP) is the most common type of primary osteoporosis, which more than half of postmenopausal females suffered from it. It is generally considered resulted in the insufficiency in endogenous estrogen by the functional degradation of ovarian. Due to the reduction of bone mass and destruction of bone microstructure caused by PMOP, patients with weak bone strength face high risks of osteoporotic fractures affecting their quality of life and leading to death in the end [1]. Not only in females, osteoporosis is likewise a serious health problem in men. In China, the incidence rate in males beyond sixty-years-old is 23% and 49% in females [2]. Because of the growing ageing population, osteoporosis as an age-related disease has become a health crisis and a definite cause of economic impact both in China and around the world. As a chronic disease, long-term treatment is needed for postmenopausal osteoporosis. However, the treatment regimens of PMOP are vastly expensive as well as related to certain side effects. For example, estrogen replacement therapy may increase the risk of breast cancer, while biphosphonates cause osteonecrosis of the jaw [3]. Hence, to reduce side effects of therapies and relieve the economic burden, Chinese herbal medicine as an inexpensive approach in clinical has been a popular alternative modality in treating postmenopausal osteoporosis.

In the long history of China, traditional Chinese medicine (TCM) is always integrated into the Chinese healthcare system and it is applied for treating various diseases. Duhuo Jisheng Decoction (DHJSD), a Tang Dynasty formula be quoted from the book Bei Ji Qian Jin Yao Fang authored by Sun Simiao, is composed of 15 Chinese herbs including Radix Angelicae Pubescentis (Du-huo), Erba Asari (Xi-xin), Radix Ledebouriellae (Fang-feng), Radix Gentianae Macrophyllae (Qin-jiao), Cortex Cinnamomi (Rou-gui), Herba Taxilli (Sang-ji-sheng), Cortex Eucommiae (Du-zhong), Radix Achyranthis Bidentatae (Niu-xi), Radix Angelicae Sinensis (Dang-gui), Radix Rehmanniae Preparata (Shu-di-huang), Rhizoma Chuanxiong (Chuan-xiong), Radix Paeoniae Rubra (Chi-shao), Radix Codonopsis (Dang-shen), Poria (Fu-ling), and Radix Glycyrrhizae (Gan-cao). In clinical practice, DHJSD can eliminate rheumatism, relieve pain, strengthen the liver and kidney, and nourish Qi and blood, which conforms to “kidney dominates bone” in the TCM theory. At present, the decoction is used primarily for osteoarthritis, and it has well anti-inflammatory activity. Some scholars believed that postmenopausal osteoporosis is a chronic inflammatory disease [4]. Thus, DHJSD treat postmenopausal osteoporosis that has theoretical backing and has been demonstrated in multiple examples [5, 6].

Therefore, here, we aimed to perform a system review through meta-analysis of DHJSD for postmenopausal osteoporosis, which center upon clinical effectiveness and medication safety.

## 2. Materials and Methods

This article is conducted as claimed by the Cochrane recommendations and was reported according to the Preferred Reporting Items for Systematic Reviews and Meta-Analyses: the PRISMA statement [7, 8].

### 2.1. Study Selection

In this system review, the criteria for including studies were as follows. All randomized controlled trials, irrespective of blinding or publication status, were enrolled. If we cannot define a study as RCT, such as there is no additional description of the randomized method, we still included the article but assessed it as being at unclear risk of bias when it comes to selection bias domain.

We excluded articles as reviews, studies on the pathogenesis or with insufficient details of outcomes. Reports in languages other than English or Chinese were excluded either.

We included participants who were the postmenopausal women with primary osteoporosis but excluded studies focusing on men or women with secondary osteoporosis. And the postmenopausal women could be diagnosed on the diagnosis of primary osteoporosis in Chinese population, which is based on bone mineral density (BMD) levels by central dual energy X-ray absorptiometry (DXA). It is defined as that BMD is 2 standard deviations (SD) or more below the peak bone mass of the ethnic origin [9].

In the experimental groups, interventions were DHJSD used singly or in combination with conventional medical therapies. And there was no limit on dosage, duration, and administration of decoction. Comparator groups could be no treatment, placebo, or conventional therapies including bisphosphonates, calcium, vitamin D, and calcitonin. While simultaneous therapies had to be the same in both of the two groups.

Primary outcomes included fracture incidence, quality of life, and death caused by osteoporosis. Secondary outcomes changed in BMD from baseline, bone biochemical indicators in blood (e.g., oestradiol (E2), bone Gla protein (BGP), alkaline phosphatase (ALP), serum calcium (Ca), and phosphorus (P)), clinical effectiveness and adverse effect or adverse drug reaction (ADR).

### 2.2. Search Strategy

Two researchers separately searched China National Knowledge Infrastructure (CNKI), WanFang, Chinese Biomedical database (CBM), Chinese VIP information, PubMed, EMBASE, Cochrane library, and Web of Science database, while the searches were accomplished from inception to July 2019. If there is disagreement between two researchers in these processes, it will come to a decision with a third party. The search strategy of PubMed listed the following, which was modified when searching other Chinese or English databases:

  #1: Osteoporosis [Mh]
  #2: Osteoporosis, Postmenopausal[Mh]
  #3: #1 or #2
  #4: Duhuo Jisheng [Tiab]
  #5: Duhuo Jisheng Decoction [Tiab]
  #6: Duhuo Jisheng Tang [Tiab]
  #7: Or/4–6
  #8: #3 and #7

Meanwhile, we identified any additional published or unpublished literature meeting the standard through handsearching reference lists of retrieved studies. The trials were integrated and deduplicated by EndNote software.

### 2.3. Data Extraction and Management

Two reviewers extracted data and characteristics relevant to analysis from studies, which included primary author, publication year, simple size of trials, patients' mean age, diagnostic criteria, administration for therapies, dosage, treatment duration, and outcome measures. Altercation could be resolved through deeper communication. After that, number and reasons for the participants who became lost-to-follow-up should be recorded too.

Two researchers following the risk of bias criteria in Cochrane Handbook evaluated the methodological quality of each trial independently. Assessment items arose from the following domains: random sequence generation, allocation concealment, blinding of participants and personnel, blinding of outcome assessment, incomplete outcome data, selective reporting, and other bias. Judgement could assign one of three levels to each item: “low” risk, “high” risk, and “unclear” risk. Altercation could be resolved by deeper discussion after consulting with another reviewer.

### 2.4. Analytical Approach

The statistical results were transferred to Review Manger 5.3 (a Cochrane software) to analyze. In this article, a dichotomous variable was presented as risk ratios (RR) with 95% confidence intervals (CI). Continuous data could be divided into 2 classes. Those with the same scale used mean difference (MD), and those with distinct scale used standardized mean difference (SMD). Both were with 95% confidence intervals. The formulation and dosage of DHJSD were different (Table 1), so heterogeneity existed. We analyzed statistical data with a random effects model. As for data unable to be merged owing to inconsistent or absent data, a descriptive analysis was presented.

In this article, all tests were two-sided unless stated otherwise. A *p* value ≤ 0.05 was regarded as statistically significant. The heterogeneity of outcomes among trials was evaluated by the Cochrane Q test and the *I*^2^ inconsistency test [10]. According to the guidance in the Cochrane book, *I*^2^ values from 0% to 40% might not be important, 40% to 60% represents moderate heterogeneity, 60% to 75% represents substantial heterogeneity, and 75% to 100% represents considerable heterogeneity. In order to seek the source of heterogeneity, we performed the data in subgroup analysis, which are based on characteristics of participants, details of the intervention, or reference standard. We would undertake a sensitivity analysis when possible. To evaluate the publication bias, funnel plots would be drawn for comparison-adjust with atleast ten trials.

### 2.5. Assessing the Quality of Evidence

Based on the GRADE recommendations, we graded the quality of evidence through GRADEpro online software (on hand in gradepro.org). In general, we assessed the evidence as four levels: high quality, moderate quality, low quality, and very low quality. Two authors assessed certainty of evidence separately, and then, they would have a discussion if there was a dispute.

## 3. Result

### 3.1. Result of the Studies

**Figure 1:**
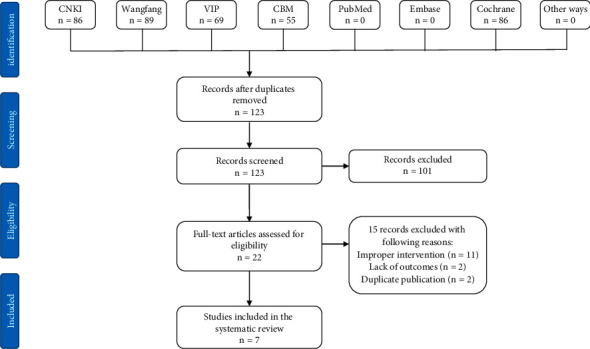
PRISMA flowchart.

Acting in accordance with the search strategy, we identified 299 references. After removal of duplicates, 123 articles remained. And then, we scanned the titles and abstracts, and 101 of these reports were excluded because of the following reasons: animal studies, traditional reviews, case reports, or multiple publication. After full-manuscript assessed, we excluded 15 records with the following reasons: improper intervention with other Chinese herbal compounds (*n* = 11), lack of outcomes (*n* = 2), and duplicate publication (*n* = 2). Eventually, 7 studies that met the criteria were contained in our review [11–17]. All the included studies were published in Chinese journals. The PRISMA statement flow chart shows this process (Figure 1).

Of the 7 articles, 8 trials were enrolled in our analysis and are described in Table 2. One article reported a three-arm experiment [16]. So, we modified it through pairwise comparisons, which mean that we enrolled it as two different trials with a same control group. A total of 650 participants were randomized into experimental groups (*n* = 325) and control groups (*n* = 325). The sample size ranged from 30 to 60. Ethnicity of all participants was Chinese. And all the studies enrolled postmenopausal woman. The variation in the mean age ranged from 44.89 to 70.85 years.

DHJSD was used singly in three trials and was plus conventional medical therapies in the other five trials. All trials chose the form of decoction, and the difference of dosage and formulation are described in Table 1. Control interventions included three types of conventional pharmaceutical medicine: alendronate, zoledronic acid, and Caltrate D.

### 3.2. Risk of Bias in Included Trials

**Figure 2:**
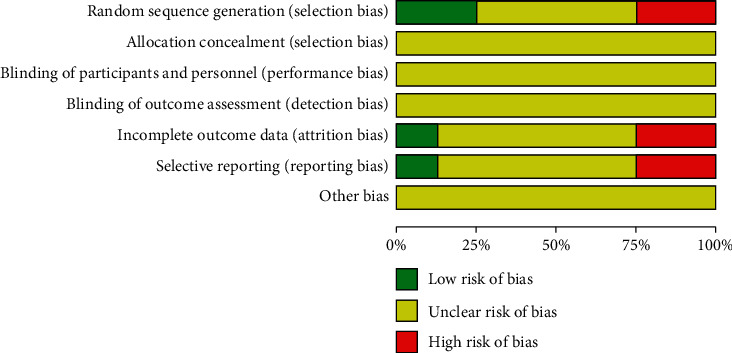
Risk of bias assessment in studies.

**Figure 3:**
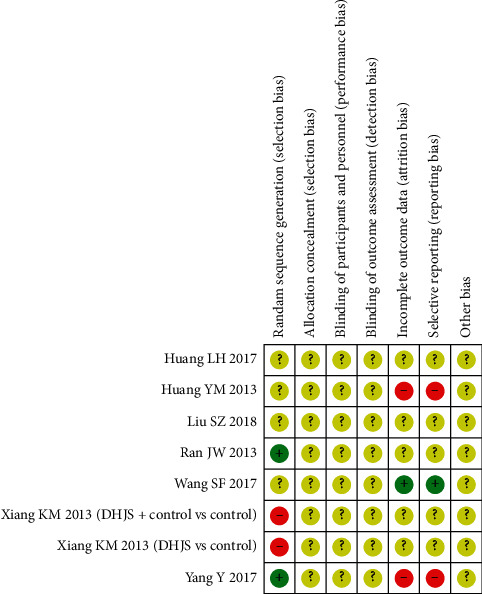
Risk of bias assessment for each included study in the review.

A summary of the risk of bias in the included trials is given in Figures 2 and 3. In this article, two trials reported the method of random numbers table when generating the allocation sequence [14, 17]. Another two trials reported the wrong method as the patients are arranged by registration order, so we assessed them as “high risk” [11, 12]. The other three trials stated the word random but had few concrete details of stochastic methods [13–15]. None of the trials provided the detail of allocation concealment. As for the blinding, only one trial mentioned single-blinding but lacked of specifics in the report [11]. The risk of incomplete outcome data could not be identified because none of the trials described patients withdraw or lost-to-follow-up. In the domain of selective reporting, we judged two trials as a high risk of selective reporting because they did not state the outcome measurement of clinical effectiveness [12, 17]. The rest of trials assessed the clinical effectiveness based on *Guidelines for the Clinical Research of Chinese Medicine New Drugs* [18]. Then, there was unclear risk of other bias across the enrolled trials on account of insufficient information. In conclusion, most of the involved experiments were deemed to have inadequate methodological quality.

### 3.3. Primary Outcomes

None of the included studies mentioned fracture incidence, quality of life, or death.

#### 3.3.1. BMD

**Figure 4:**
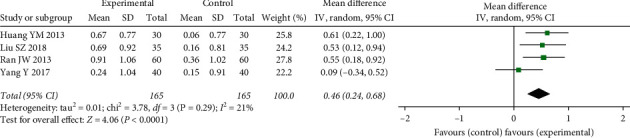
Forest plot of DHJSD vs. conventional therapies on BMD of the lumbar spine.

*(1) BDM of the Lumbar Spine*. Five studies showed the effects of DHJSD on BDM levels in the lumbar spine among the patients. Due to difference in the formulation and dosage of DHJSD, heterogeneity existed. And the pooled consequences showed a high heterogeneity across all these trials (*I*^2^ = 60%, *P*=0.02). Then, we performed sensitivity analyses based on the duration of administration. We enrolled four RCTs including 165 participants over a three-month treatment. A pooled result indicated a significant increase of the lumbar spine BDM with DHJSD, when compared to conventional therapies (MD: 0.46 (0.24, 0.68), *P* < 0.0001; *I*^2^ = 21%) (Figure 4).

#### 3.3.2. Bone Biochemical Indicators

*(1) Estradiol*. The change in serum estradiol (E2) in participants treated by DHJSD or DHJSD plus cointervention versus conventional supplementation (i.e., zoledronic acid, alendronate, and Caltrate D) for 3 and 12 months was described in 6 RCTs. And they included 235 PMOP patients totally.

**Figure 5:**
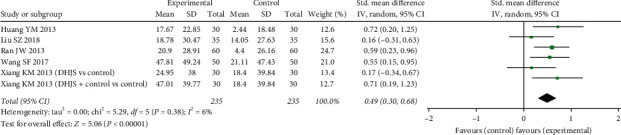
Forest plot of DHJSD vs. conventional therapies on improving blood E2.

Due to the difference in the formulation and dosage of DHJSD, heterogeneity existed. We used the random effect model to pool data. The overall data showed a statistically significant increase of serum estradiol levels in experimental groups (SMD: 0.49 (0.30, 0.68), *P* < 0.0001; *I*^2^ = 6%) (Figure 5).

**Figure 6:**
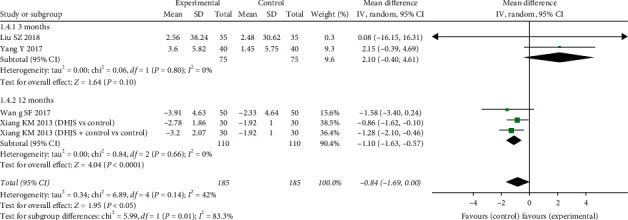
Forest plot of DHJSD vs. conventional therapies on changing blood BGP.

*(2) Bone Gla Protein*. Five trials evaluated the effects of DHJSD plus western medical cares (or DHJSD alone) versus the same western medical cares that included zoledronic acid, alendronate, and Caltrate D [13, 15–17]. The meta-analyses showed no significant clinic effect for changing the level of BGP (MD = −0.84 (−1.69, 0.00), *P* = 0.05). But there was a moderate heterogeneity across enrolled studies due to the difference in the formulation and dosage of DHJSD and *P* = 0.14, *I*^2^ = 42%. Subsequently, the sensitivity analyses were conducted according to the course of treatments. Two trials observed the change of BGP in blood after 3 months [13, 17]. The analyses indicated there was no significant difference in BGP for the DHJSD treatment groups (MD:2.10 (−0.40, 4.61), *P* = 0.01; *I*^2^ = 0%). The rest of trials had a twelve-month treatment duration [15, 16]. And the integrated data illustrated a significant reduction in BGP for those treated with DHJSD (MD = −1.10 (−1.63, −0.57), *P* < 0.0001; *I*^2^ = 0%) (Figure 6).

#### 3.3.3. Clinical Effective Rate

**Figure 7:**
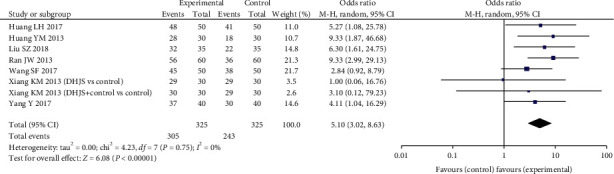
Forest plot of DHJSD vs. conventional therapies on improving clinical effective rate.

Results on the rate of clinical effectiveness were presented in eight trials involving 600 PMOP patients. In the analysis, outcome variable of this item is measured in dichotomous form. The analysis indicated that those treated by DHJSD had a statistically significant augment in clinical effectiveness (RR: 1.23 (1.06, 1.42), *P* = 0.006). But due to difference in the formulation and dosage of DHJSD, there was a significant heterogeneity (*P* < 0.0001, *I*^2^ = 85%). Then, we performed sensitivity analyses through expressing outcomes as odd risk (OR). The pooled data showed a significant positive effect of experimental groups compared with the conventional therapy groups (OR: 5.07 (3.07, 8.35), *P* < 0.0001; *I*^2^ = 0%) (Figure 7).

#### 3.3.4. Adverse Effect

Only one trial touched upon adverse events associated with DHJSD treatment [15]. Few patients experienced some mild stomach discomfort such as nausea and vomiting. The other seven trials did not report this item.

#### 3.3.5. Publication Bias

As we discussed above, the publication bias could not be assessed because of the deficiency trials data.

#### 3.3.6. Quality of Evidence

We assessed the quality of evidence in the current meta-analysis. A part of the evidence was in a low level, such as BMD of the lumbar spine and femoral neck, and E2. The other enroll items were very low. The results of evaluation were not satisfied generally. Indirectness and risk of bias were the salient problem of the including data. Moreover, few upgraded factors could be identified. The details are summarized in Table 3.

## 4. Discussion

Traditional herbal medicines have been used as complementary and alternative treatment options for osteoporosis patients for a long time. Some reviews have assessed the efficacy and safety of different Chinese multiherb prescriptions in treating osteoporosis, such as Liuwei Dihuang decoction and Erxian decoction [19, 20]. But considering the diversity of Chinese herbal medicine, we have reason to believe that there is still huge part of diverse herbs compound recipes which remains unexplored. To our knowledge, this is the first systematic review evaluating the efficacy and side effects on DHJSD for PMOP that has been reported.

The pooled estimate suggested that there is a positive effect in increasing BMD of the lumbar spine after 3 months in four RCTs. The patients derived a significant clinical benefit in E2 when DHJSD treatment ongoing in 6 trials and 4 of the enroll trials used DHJSD plus conventional therapies. In serum BGP levels, three studies showed significant effects in attenuating the content of BGP after twelve-month treatment, while there were no significant differences in two three-month experiments. There is no significant difference in benefit regarding other bone biochemical indicators such as ALP, Ca, or P. The current analysis showed a significant benefit of DHJSD treatment in regard of clinical effectiveness overall. The adverse effect of DHJSD might be some mild stomach upset. It seemed that DHJSD make some mitigation of symptom in PMOP patients but in unconfirmed safety.

In the clinic, DHJSD is usually used for lumbar disc herniation, ankylosing spondylitis, and knee osteoarthritis. It shows several pharmacological activities such as anti-inflammatory, analgesic, and antitumor. As for the bone metabolic, prior studies have shown that Duhuo Jisheng decoction can weaken osteoclast differentiation and function through inhibition of RANKL in osteoblast cells [21]. Our results give evidence for the probable mechanism of DHJSD as a treatment for PMOP. It has been showed that treatment with antibone-resorption agents might result in the decrease of the BGP level, whereas the therapy to promote bone formation can make the BGP level increasing [22]. Experimental data showed that there was a significant decrease in the BGP level after a twelve-month treatment, suggesting that DHJSD treats postmenopausal osteoporosis via inhibiting bone resorption. Furthermore, DHJSD has been declared to have a kind of pharmacological effects in inhibiting inflammatory factors such as TNF-*α* and IL-1*β*, which are key factors for osteoclast maturation [23]. It has also previously observed that estrogen deficiency make inflammatory factors such as TNF-*α* upregulated, which enhances osteoclast expression and activity, and eventually results in bone resorption [24]. Another pooled data in current meta-analysis showed that DHJSD can increase serum E2 levels significantly, especially in combined western therapies groups. Taken together, our review indicated that DHJSD may be an optional therapy for postmenopausal osteoporosis by increasing the serum E2 levels to inhibit inflammatory factors, in turn, attenuating bone resorption. However, more experiments need to be further conducted in order to validate this mechanism.

However, findings above may be somewhat limited by the poor methodological quality and small sample sizes of the trials identified in the review. First, none of the trials set the placebo-controlled group, and the control groups were under different treatments. Lacking randomization details were a serious issue for most of the included studies. They always lost sight of reporting methods of randomization or conducted with a wrong method such as grouped in order of visitation. And very few studies mentioned allocation concealment. Second, due to the characteristic of Chinese herbal medicines, implementing blinding can be difficult during experiment, which may lead to a bias of the intended intervention. Also, inadequate information of lost-to-follow-up patients was found in previous articles. What is more, we could not observe the primary outcomes such as quality of life or fracture incidence. Endpoint outcomes from original research studies were not direct enough. It is possible that results of clinical effectiveness are biased, given the self-reported nature of the questionnaire method. The data of relevant endpoints with potential risk of bias was given more prominence when conducting meta-analyses. Those problems lead to the risk of bias in current meta-analysis, and overall evidence remains poor generally. To sum up, it is premature to draw a conclusion whether DHJSD has a wide safety range in different treatment modalities and settings.

## 5. Conclusion

Based on this meta-analysis, it suggests that DHJSD monotherapy or DHJSD plus antiosteoporosis drugs may have beneficial effects for PMOP in the following aspects: the improvement of BMD at the lumbar spine, serum estradiol levels, and clinical effectiveness. However, a definite conclusion on other indicators and safety cannot be drawn from existing evidence. More high-quality and strict studies with large samples are needed to reassess or confirm current results.

## Figures and Tables

**Table 1:** Composition of treatment formula.

Study ID	Composition of treatment formula
Lehui [11]	Radix Angelicae Pubescentis (Du-huo) 6 g, Erba Asari (Xi-xin) 3 g, Radix Ledebouriellae (Fang-feng) 6 g, Radix Gentianae Macrophyllae (Qin-jiao) 12 g, Cortex Cinnamomi (Rou-gui) 2 g, Herba Taxilli (Sang-ji-sheng) 18 g, Cortex Eucommiae (Du-zhong) 12 g, Radix Achyranthis Bidentatae (Niu-xi) 6 g, Radix Angelicae Sinensis (Dang-gui) 12 g, Radix Rehmanniae Preparata (Shu-di-huang) 15 g, Rhizoma Chuanxiong (Chuan-xiong) 6 g, Radix Paeoniae Alba (Bai-shao) 10 g, Radix Codonopsis (Dang-shen) 12 g, Poria (Fu-ling) 12 g, and Radix Glycyrrhizae (Gan-cao) 3 g
Yumei [12]	Radix Angelicae Pubescentis (Du-huo), Erba Asari (Xi-xin), Radix Ledebouriellae (Fang-feng), Radix Gentianae Macrophyllae (Qin-jiao), Cortex Cinnamomi (Rou-gui), Herba Taxilli (Sang-ji-sheng), Cortex Eucommiae (Du-zhong), Radix Achyranthis Bidentatae (Niu-xi), Radix Angelicae Sinensis (Dang-gui), Radix Rehmanniae Preparata (Shu-di-huang), Rhizoma Chuanxiong (Chuan-xiong), Radix Paeoniae Rubra (Chi-shao), Radix Codonopsis (Dang-shen), Poria (Fu-ling), and Radix Glycyrrhizae (Gan-cao). Not report dosage form.
Shangzhi and Juntao [13]	Radix Angelicae Pubescentis (Du-huo) 10 g, Erba Asari (Xi-xin) 3 g, Radix Ledebouriellae (Fang-feng) 10 g, Radix Gentianae Macrophyllae (Qin-jiao) 12 g, Cortex Cinnamomi (Rou-gui) 6 g, Herba Taxilli (Sang-ji-sheng) 12 g, Cortex Eucommiae (Du-zhong) 15 g, Radix Achyranthis Bidentatae (Niu-xi) 15 g, Radix Angelicae Sinensis (Dang-gui) 12 g, Radix Rehmanniae Preparata (Shu-di-huang) 12 g, Rhizoma Chuanxiong (Chuan-xiong) 12 g, Paeoniae Rubra (Chi-shao) 10 g, Radix Codonopsis (Dang-shen) 15 g, Poria (Fu-ling) 15 g, and Radix Glycyrrhizae (Gan-cao) 10 g
Jinwei et al. [14]	Radix Angelicae Pubescentis (Du-huo) 15 g, Erba Asari (Xi-xin) 3 g, Radix Ledebouriellae (Fang-feng) 20 g, Radix Gentianae Macrophyllae (Qin-jiao) 15 g, Cortex Cinnamomi (Rou-gui) 6 g, Herba Taxilli (Sang-ji-sheng) 15 g, Cortex Eucommiae (Du-zhong) 15 g, Radix Achyranthis Bidentatae (Niu-xi) 15 g, Radix Angelicae Sinensis (Dang-gui) 15 g, Radix Rehmanniae Preparata (Shu-di-huang) 6 g, Rhizoma Chuanxiong (Chuan-xiong) 6 g, Paeoniae Rubra (Chi-shao) 6 g, Radix Codonopsis (Dang-shen) 6 g, Poria (Fu-ling) 9 g, and Radix Glycyrrhizae (Gan-cao) 12 g
Shaofeng [15]	Radix Angelicae Pubescentis (Du-huo) 9 g, Erba Asari (Xi-xin) 6 g, Radix Ledebouriellae (Fang-feng) 6 g, Radix Gentianae Macrophyllae (Qin-jiao) 6 g, Cortex Cinnamomi (Rou-gui) 6 g, Herba Taxilli (Sang-ji-sheng) 6 g, Cortex Eucommiae (Du-zhong) 6 g, Radix Achyranthis Bidentatae (Niu-xi) 6 g, Radix Angelicae Sinensis (Dang-gui) 6 g, Radix Rehmanniae Preparata (Shu-di-huang) 6 g, Rhizoma Chuanxiong (Chuan-xiong) 6 g, Paeoniae Rubra (Chi-shao) 6 g, Radix Codonopsis (Dang-shen) 6 g, Poria (Fu-ling) 6 g, and Radix Glycyrrhizae (Gan-cao) 6 g
Keming et al. [16]	Radix Angelicae Pubescentis (Du-huo) 9 g, Erba Asari (Xi-xin) 6 g, Radix Ledebouriellae (Fang-feng) 6 g, Radix Gentianae Macrophyllae (Qin-jiao) 6 g, Cortex Cinnamomi (Rou-gui) 6 g, Herba Taxilli (Sang-ji-sheng) 6 g, Cortex Eucommiae (Du-zhong) 6 g, Radix Achyranthis Bidentatae (Niu-xi) 6 g, Radix Angelicae Sinensis (Dang-gui) 6 g, Radix Rehmanniae Preparata (Shu-di-huang) 6 g, Rhizoma Chuanxiong (Chuan-xiong) 6 g, Paeoniae Rubra (Chi-shao) 6 g, Radix Codonopsis (Dang-shen) 6 g, Poria (Fu-ling) 6 g, and Radix Glycyrrhizae (Gan-cao) 6 g
Yang et al. [17]	Radix Angelicae Pubescentis (Du-huo) 15 g, Lycii Fructus (Gou-qi-zi) 20 g, Radix Ledebouriellae (Fang-feng) 15 g, Herba Taxilli (Sang-ji-sheng) 20 g, Cortex Eucommiae (Du-zhong) 15 g, Corni Fructus (Shan-zhu-yu) 15 g, Testudinis Carapax et Plastrum (Gui-jia) 15 g, Colla Cornus Cervi (Lu-jiao-jiao) 15 g, Dipsaci Radix (Xu-duan) 15 g, Semen Cuscutae (Tu-si-zi) 15 g, Radix Achyranthis Bidentatae (Niu-xi) 15 g, Radix Rehmanniae Preparata (Shu-di-huang) 15 g, Flos Carthami (Hong-hua) 10 g, Rhizoma Chuanxiong (Chuan-xiong) 10 g, Paeoniae Rubra (Chi-shao) 6 g, and Radix Glycyrrhizae (Gan-cao) 5 g

**Table 2:** Characteristics of the included trials.

Study ID	Sample	Age (years, mean)		Interventions		Duration	Outcomes
	Size (T/C)	T	C	T	C	In months	
Lehui [11]	100 (50/50)	54.3 ± 3.2	54.8 ± 3.5	DHJSD	Alendronate (10 mg, qd, po)	3	①
Yumei [12]	60 (30/30)	64.0 ± 6.2	65.0 ± 3.3	DHJSD + control	Zoledronic acid (4 mg, Q20 d, ivgtt)	3	①, ②, (a), ④, ⑤, and ⑥
Shangzhi and Juntao [13]	70 (35/35)	63.0 ± 3.3	64.0 ± 6.2	DHJSD	Alendronate (70 mg, qw, po)	3	①, ②, (a), ③, and ④; NBAP
Jinwei et al. [14]	120 (60/60)	67 ± 5	66 ± 6	DHJSD + control	Caltrate D (600 mg, qn, po)	3	①, ②, (a, b), ④, ⑤, ⑥, and ⑦
Shaofeng [15]	100 (50/50)	58.87 ± 11.08	58.64 ± 12.21	DHJSD + control	Zoledronic acid (5 mg, -, ivgtt)	12	①, ②, ③, and④
Keming et al. [16]	60 (30/30)	57.3	57.3	A : DHJSD + control	C: zoledronic acid (4 mg, Q30 d, ivgtt)	12	①, ②, (a), ③, ④, and ⑦
Keming et al. [16]	60 (30/30)	57.3	57.3	B : DHJSD	C: zoledronic acid (4 mg, Q30 d, ivgtt)	12	①, ②, (a), ③, ④, and ⑦
Yang et al. [17]	80 (40/40)	53.69 ± 7.76	52.82 ± 7.93	DHJSD + control	Caltrate D (600 mg, 1-2 tablets, qn, po),	3	①, ②, (a, b), and ③; CTX

*Note.* ①, clinical effectiveness; ②, BMD (a, lumbar spine and b, femoral neck); ③, BGP; ④, E2; ⑤, P; ⑥, Ca; and ⑦, ALP, the information is lost; NBAP, bone alkaline phosphatase; CTX, c-terminal crosslinking telopeptide.

**Table 3:** Summary of findings tables of DHJSD for PMOP.

Outcomes	Anticipated absolute effects (95% CI)		Relative effect (95% CI)	Participants (studies)	Certainty of the evidence (GRADE)
	Risk with conventional therapies	Risk with Duhuo Jisheng decoction			
	Study population				
Clinical effective rate	748 per 1,000	920 per 1,000 (793–1,000)	RR 1.23 (1.06, 1.42)	650 (8 RCTs)	⊕⊝⊝⊝ Very low
BDM of the lumbar spine	The mean BDM of the lumbar spine was 0	MD 0.46 higher (0.24 higher–0.68 higher)	—	330 (4 RCTs)	⊕⊕⊝⊝ Low
BGP	The mean BGP was 0	MD 0.84 lower (1.69 lower–0)	—	370 (5 RCTs)	⊕⊝⊝⊝ Very low
E2	The mean E2 was 0	SMD 0.49 higher (0.3 higher–0.68 higher)	—	470 (6 RCTs)<	⊕⊕⊝⊝ Low

⊕ means high quality, and ⊖ means low quality. These two symbols come from the GRADE system. They are used to classify the certainty of evidence. For a piece of evidence, the more the patient-important outcomes we are confident, the more the symbol “⊕” will be show. The GRADE system classifies the certainty of evidence as follows: high further research is very unlikely to change our confidence in the estimation of effect. Moderate further research is likely to have an important impact on our confidence in the estimation of effect and may change the estimate. Low further research is very likely to have an important impact on our confidence in the estimation of effect and is likely to change the estimate. Very low means any estimate of effect is very uncertain.

## Data Availability

The data used to support this study are included within this article.
